# Changes in equine strongylid communities after two decades of annual anthelmintic treatments at the farm level

**DOI:** 10.1007/s00436-024-08417-5

**Published:** 2024-11-25

**Authors:** Tetiana A. Kuzmina, Alžbeta Königová, Anatoliy Antipov, Yuriy Kuzmin, Vitaliy Kharchenko, Yaroslav Syrota

**Affiliations:** 1 Institute of Parasitology, Slovak Academy of Sciences, Hlinkova 3, Kosice, Slovakia; 2 I. I. Schmalhausen Institute of Zoology NAS of Ukraine, B. Khmelnytsky Street, 15, Kiev, Ukraine; 3 Bila Tserkva National Agrarian University, Vul. Stavyshanska, 128, Bila Tserkva, Ukraine

**Keywords:** Cyathostomins, Parasite community structure, Species richness, Species diversity, Strongylid community, Horses

## Abstract

The aim of this study was to assess the changes in strongylid communities of domestic horses after two decades of regular anthelmintic treatments; the changes in prevalence and relative abundance of individual strongylid species and their contribution to the observed alterations were estimated. The study was conducted in 2023; data collected in 2004 were used for comparison; 39 horses from two farms in Ukraine (22 horses in 2004 and 17 in 2023) were examined. In total, 18,999 strongylid specimens were collected by in vivo diagnostic deworming method before (in 2004, 9119 specimens) and after (in 2023, 9880 specimens) frequent application of anthelmintics. Strongylids were identified morphologically. Thirteen strongylid species were found in horses in 2023; only small strongylids (Cyathostominae) were recorded. In 2004, 21 species were found: 6 species of large strongylids (Strongylinae) and 15 of cyathostomins. Species richness (Margalef’s index) and species diversity (Shannon’s and Simpson’s indexes) decreased over two decades on both farms; the Berger-Parker dominance index for *C. nassatus* dramatically increased up to 74.4. The dissimilarity of strongylid communities of 2023 and 2004 was primarily connected with the disappearance of large strongylids and rare cyathostomin species after two decades of regular anthelmintic treatments. SIMPER analysis revealed that *C. nassatus* and *C. catinatum* mostly contributed to this dissimilarity. A trend of gradual transformation of the strongylid community structure from multimodal (in 2004) to bimodal (in 2023) was observed on both farms.

**Supplementary Information:**

The online version contains supplementary material available at 10.1007/s00436-024-08417-5.

## Introduction

Nematodes of the family Strongylidae are the most common and prevalent helminth parasites of wild and domestic equids; they infect up to 100% of horses worldwide (Lichtenfels et al. 2008; Kuzmina et al. 2016; Bellaw and Nielsen 2020; Jürgenschellert et al. 2022). Strongylids have the highest species diversity among the horse parasites. So far, 64 strongylid species have been described, including 14 species of large strongylids (subfamily Strongylinae) and 50 species of small strongylids (subfamily Cyathostominae) (Lichtenfels et al. 2008). Additionally, several cryptic species of cyathostomins have been detected by molecular methods (Hung et al. 1999; Bredtmann et al. 2019; Louro et al. 2021).

Typically, up to more than 20 strongylid species may simultaneously parasitize an individual horse (Reinemeyer et al. 1984; Mfitilodze and Hutchinson 1990; Kuzmina et al. 2016; Bellaw and Nielsen 2020) forming a complex and diverse community in the horse large intestine (Kuzmina et al. 2007, 2011, 2016; Sallé et al. 2018; Boisseau et al. 2023). Strongylids are also considered one of the most pathogenic groups of equine parasites, which cause severe health problems that may result in the death of horses, especially of young foals (Ogbourne 1978; Herd 1990; Love et al. 1999; Lyons et al. 1999; Nielsen 2012; Pfister and van Doorn 2018; Nielsen and Reinemeyer 2018).

Nowadays, the primary method of strongylid control is the use of anthelmintic drugs (Kaplan and Nielsen 2010; Nielsen et al. 2013; Nielsen 2022). Since the 1960s, when benzimidazole (BZ) anthelmintics became widely available on the market, interval-based treatment programs based on routine and frequent administration of anthelmintics to entire horse populations in a prophylactic manner have been used extensively in the equine industry worldwide (Drudge and Lyons 1966; Matthews 2014; Nielsen 2022). This extensive approach led to the development of resistance to BZs and other groups of anthelmintics in horse parasitic nematodes (Kaplan 2004; Matthews 2008, 2014; Peregrine et al. 2014; Nielsen 2022). Frequent use of anthelmintics and, especially, macrocyclic lactones (ML) significantly changed the structure of the strongylid communities in horses (Kuzmina et al. 2016; Abbas et al 2024a, b) and could promote the selection of drug-resistant strains in some species.

Studies addressing strongylid species diversity in wild and domestic equids have been ongoing for over a century (Lichtenfels et al. 2008). A meta-analysis of data collected from domestic horses over 50 years has shown that a small number (5–10) of cyathostomin species dominate the strongylid communities in horses worldwide. Among these species, *Cylicocyclus nassatus*, *Cylicostephanus longibursatus*, and *Cyathostomum catinatum* are the most abundant (Bellaw and Nielsen 2020). Various factors such as age and breed of horse and the frequency of anthelmintic treatments have been reported to influence the strongylids and their community structure (Kornaś et al. 2010; Saeed et al. 2010; Kuzmina et al. 2016; Slivinska et al. 2016; Sallé et al. 2018; Boelow et al. 2023). However, detailed studies focusing on the impact of anthelmintic treatments on individual strongylid species and the structure of their communities have not been carried out due to the difficulties of collecting and identifying these nematodes. Application of the new nemabiome approach was elaborated for determining the presence of the strongylid species parasitized horses and assessing their populations (Poissant et al. 2021; Sargison et al. 2022; Courtot et al. 2023; Halvarsson et al. 2024). However, this approach still does not allow for accurate estimation of the parameters of the strongylid community.

Our long-term research on the species diversity and structure of strongylid communities in domestic and wild equids performed in Ukraine has revealed some alterations in the strongylid communities caused by frequent use of anthelmintics (Kuzmina et al. 2005, 2007, 2016, 2020; Kuzmina and Kharchenko 2008). Nevertheless, none of these studies included long-term observations of individual farms. Recently, we had the opportunity to repeat the collection of strongylids from two farms where we had previously collected samples in 2004–2006 (Kuzmina et al. 2005, 2016) using the same collection methods. These new data allowed us to compare the strongylid communities on two farms over two decades, with each farm analyzed separately. To our knowledge, it is the first study of equine strongylids with such a design. This study aimed to assess the changes in strongylid communities of domestic horses after two decades of regular anthelmintic treatments. Specifically, it focused on analyzing the changes in prevalence and relative abundance of individual strongylid species over this period and determining the contribution of each species to the observed alterations.

## Material and methods

### Experimental design: farms and horses studied

The study was conducted in September and November 2023 at two horse farms from the Kyiv region, Ukraine (Farms #1 and #2). Farm #1 had 40 Ukrainian saddlers used for sport, breeding, and recreational riding; horses were housed in stables and had limited access to a permanent pasture (about 20 ha) for 6–14 h daily depending on the season during the whole year. At this farm, regular deworming of all horses 2–3 times per year using macrocyclic lactone (ML) drugs has been practiced since 2005. Nine horses 2–14 years old from Farm #1 were selected for our study. Farm #2 had 18 mixed-breed horses used for recreational riding and breeding; the horses were housed in stables and had limited access to a large permanent paddock (about 5 ha) for 6–10 h daily. At this farm, regular deworming of all horses with various BZ and ML anthelmintics twice a year has been practiced since 2010. Eight horses 3–11 years old from Farm #2 were selected for our study. All information about age, breed, management (horse-keeping conditions, stocking density, stable cleaning, access to pasture, quarantine for newly arrived horses, etc.), and deworming of the horses was obtained from the horse owners.

For comparison, the original datasets on the strongylid species collected in 2004 from Farm #1 (10 horses) and Farm #2 (12 horses) (Kuzmina and Kharchenko 2008) were used. The experimental design and method of nematode collection were identical in the studies performed in 2004 and 2023. According to information from horse owners, Farm #1 had the same horse-management practice for two decades, except for the anthelmintic treatment strategy. Before 2005, horses were treated occasionally < 1–2 times a year only with BZ anthelmintics. Farm #2 also had the same horse-management conditions since 2004; before our study in 2004, no anthelmintics had been used on the farm for more than 4 years.

### Experimental design: collection of strongylids

Strongylid nematodes were collected from all horses using an in vivo diagnostic deworming method (Kuzmina et al. 2005). Fecal egg count (FEC) using the McMaster technique (Herd 1992) with a sensitivity of 25 eggs per gram of feces (EPG) was performed for every horse individually before treatment. Only horses with EPG > 200 were selected for the study. All selected horses were treated with the macrocyclic lactone drug “Nemasectin” (1% aversectin C, UkrZooVetPrompostach, Ukraine). Fecal sampling was performed 24, 36, 48, and 60 h after treatment; the fecal samples were washed with 0.9% saline and examined under a magnifying glass to collect strongylids. Because of the large number of samples collected simultaneously, to avoid the destruction of nematodes, some samples were frozen (at the temperature − 18 °C) for a couple of days/weeks before processing. All strongylids expelled were collected manually and fixed in 70% ethanol. Fecal egg counts using the McMaster technique were performed 14 days after treatment; no strongylid eggs were detected in any samples; thus, all intestinal-lumen stages of strongylids were expelled from the horse intestines and available for morphological examination. Before identification, all strongylids were clarified in lactophenol (25% lactic acid, 25% phenol, 25% glycerin, 25% distilled water) and identified under a light microscope by morphological criteria (Lichtenfels et al. 2008). In total, 9880 strongylid specimens were collected, identified, and used for the strongylid community structure analysis.

### Data analysis

Data summaries and descriptive analyses were calculated using Excel for Microsoft 365 (Version 2311). For each strongylid species, the prevalence (*P*) of infection was calculated as suggested by Bush et al. (1997) in each sample. However, in our study, the concept of prevalence was somewhat different from the definition of Bush et al. (1997), as it was calculated only for a selected sample of horses (with EPG > 200) and described the occurrence of each strongylid species on the farm. The proportion of each species in the strongylid community, or the relative abundance (RA) of the species, was estimated as the percentage of all specimens of the species in the whole helminth number in the helminth component community. The distribution of collected strongylid species into ten abundance classes was carried out according to Bucknell et al. (1996).

Comparative analysis of the strongylid communities on the two farms over two decades was performed using the datasets including 18,999 strongylid specimens; among them, 9880 specimens were collected in 2023, and 9119 specimens were collected in 2004 as the original dataset of the previous study (Kuzmina and Kharchenko 2008).

Similarities of the strongylid species composition between component communities from farms and years were calculated using the Jaccard index (J). All species diversity indexes (Margalef’s species richness index, Pielou’s evenness index, Berger-Parker dominance index, Shannon’s and Simpson’s diversity indexes) were calculated in PAST 3.1 software (Hammer 1999–2015). The contribution of the individual strongylid species to the dissimilarity between the strongylid communities of 2004 and 2023 was identified using the SIMPER routine implemented in PRIMER 6 (Clarke and Gorley 2006). For each species recorded in both farms, a comparison of the prevalence was performed by the unconditional exact test (Reiczigel et al. 2008) using Quantitative Parasitology (QP) v. 3.0 computer program (Rózsa et al. 2000).

The following approach was used to compare helminth species’ relative abundances for each farm between 2004 and 2023. Initially, only species present in both years were selected for comparison. The relative abundances and their confidence intervals were then calculated for each species. The 95% confidence intervals were calculated with the function *binom.bayes* from the package “binom” (Dorai-Raj 2022) installed in the R environment (R core Team 2023). The calculations used a binomial Bayesian model, specifying the number of positive cases and total cases for each group. The central type of estimation was applied, with prior distributions set using a Beta(1,1). Finally, only species with confidence intervals that did not overlap were considered to have essentially changed in relative abundance between the two years, and the data were visualized as bar plots.

To visualize changes in helminth communities between 2004 and 2023, non-metric multidimensional scaling (nMDS) ordinations were performed on the helminth abundance matrices for each farm. At first, the data underwent square-root and Wisconsin transformations for standardization. The Bray–Curtis dissimilarity index was used to calculate the differences between every helminth infracommunities in each dataset. The nMDS calculations were conducted using the *metaMDS* function from the “vegan” package (Oksanen et al. 2022). The datasets with the obtained nMDS coordinates of individual horses were combined with data from the years (2004 or 2023) when each horse was sampled. Finally, the data were visualized as scatter plots.

The relationship between the number of nematodes collected per horse and species richness — the number of nematode species found per horse — was estimated using a Poisson generalized linear model (GLM) (Supplement 1). The regression model was fitted with species richness as the response variable and the number of nematodes collected per horse as the predictor. A visualization of the effect of species richness on sampling effort was done with the “visreg” package (Breheny and Burchett 2017).

Data manipulation and most visualization procedures were conducted using the “tidyverse” collection of packages (Wickham et al. 2019).

## Results

### Species composition of the strongylid communities

Thirteen strongylid species were found in horses from two farms in 2023; 12 species in Farm #1 and 9 species in Farm #2. From 4 to 10 species parasitize a horse; the number of strongylid species found per host varied between farms (Table 1). Only small strongylids (subfamily Cyathostominae) were detected on both farms. In contrast, in the samples collected in 2004, 21 strongylid species were recorded in two farms: six species of large strongylids (subfamily Strongylinae) and 15 species of cyathostomins. From 7 to 19 species parasitized each horse; the number of species also varied between farms (Table 1). The regression analysis did not reveal a significant effect of the number of nematodes collected in a sample from a horse on species richness (*b* = 0.0001, *z* = 0.892, *p* = 0.372) (Supplement 2).

**Table 1:** Prevalence (*P*, %) and relative abundance (RA, %) of strongylid species found in horses on Farms #1 and #2 over two decades. The species whose prevalence increased/decreased significantly (based on the unconditional exact test, *p* < 0.05) are marked in bold

Species		Farm #1				Farm #2			
		2004		2023		2004		2023	
		*P*, %	RA, %	*P*, %	RA, %	*P*, %	RA, %	*P*, %	RA, %
Subfamily Strongylinae									
1	*Strongylus vulgaris*	8.3	0.1	—	—	58.3	1.5	—	—
2	*S. edentatus*	—	—	—	—	66.7	0.4	—	—
3	*S. equinus*	—	—	—	—	41.7	0.2	—	—
4	*Triodontophorus serratus*	16.7	0.2	—	—	58.3	0.2	—	—
5	*T. brevicauda*	8.3	0.1	—	—	33.3	0.3	—	—
6	*T. nipponicus*	—	—	—	—	33.3	0.4	—	—
SubfamilyCyathostominae									
7	*Cyathostomum catinatum*	100.0	23.5	88.9	2.3	100.0	6.4	100.0	19.8
8	*C. pateratum*	83.3	6.4	77.8	11.5	8.3	< 0.1	—	—
9	*Coronocyclus coronatus*	66.7	1.8	66.7	1.3	83.3	1.3	75.0	5.9
10	*C. labiatus*	8.3	< 0.1	11.1	< 0.1	25.0	0.5	12.5	< 0.1
11	*C. labratus*	25.0	0.3	11.1	0.1	83.3	3.1	—	—
12	***Cylicostephanus calicatus***	**100.0**	**6.2**	**33.3**	**0.3**	91.7	6.2	87.5	19.6
13	***C. minutus***	**25.0**	**0.2**	**77.8**	**0.5**	83.3	8.5	—	—
14	***C. longibursatus***	91.7	4.9	100.0	4.9	**91.7**	**18.9**	**50.0**	**18.3**
15	*C. goldi*	91.7	4.1	100.0	4.4	75.0	0.7	87.5	1.1
16	*C. bidentatus*	8.3	0.1	—	—	—	—	—	—
17	*Cylicocyclus nassatus*	100.0	28.8	100.0	74.4	100.0	27.6	100.0	30.6
18	***C. ashworthi***	83.3	5.3	66.7	0.2	**91.7**	**4.5**	**12.5**	**4.1**
19	*C. leptostomus*	66.7	17.2	22.2	0.1	91.7	15.8	—	—
20	***C. insigne***	66.7	0.4	—	—	**75.0**	**0.9**	**25.0**	**0.6**
21	*C. elongatus*	—	—	—	—	8.3	< 0.1	—	—
22	*C. ultrajectinus*	8.3	0.1	—	—	—	—	—	—
23	*Parapoteriostomum mettami*	16.7	0.3	—	—	—	—	—	—
24	*Petrovinema poculatum*	—	—	—	—	8.3	2.5	—	—
Number of species per horse, mean (min–max)		9.8 (7–15)		7.4 (6–10)		13.8 (7–19)		6.4 (4–8)	

A direct comparison of the species composition in samples collected in 2023 and 2004 revealed that dissimilarity between samples from the farms was primarily due to the disappearance of large strongylids from the communities after two decades (Table 1). Also, rare cyathostomin species (with *P* < 20%) (*C. elongatus*, *C. ultrajectinus*, *P. mettami*, *P. poculatum*, *C. bidentatus*) disappeared from the communities; the prevalence of the background species (with *P* = 20–50%) (*C. labiatus*, *C. labratus*) dramatically decreased in 2023, and these species became rare, or disappeared, as *C. insigne* had.

### Changes in the prevalence and relative abundance of strongylid species

Changes in the prevalence of individual strongylid species in the farms over two decades (Table 1) allowed us to distinguish three categories of species: category 1, species whose prevalence did not change significantly (*C. nassatus*, *C. catinatum*, *C. coronatus*, *C. goldi* on both farms, and *C. longibursatus* on Farm #1); category 2, species whose prevalence changed significantly (based on the unconditional exact test, *p* < 0.05) (*C. calicatus* and *C. minutus* on Farm #1, *C. ashworthi*, *C. longibursatus,* and *C. insigne* on Farm #2) (see Table 1); category 3, species that disappeared from the community (all species of the subfamily Strongylinae, *C. bidentatus*, *C. elongatus*, *C. ultrajectinus*, *P. mettami*, *P. poculatum*, *C. labratus*, *C. pateratum*, *C. insigne* on Farm #1, and *C. leptostomus* on Farm #2).

**Fig. 1:**
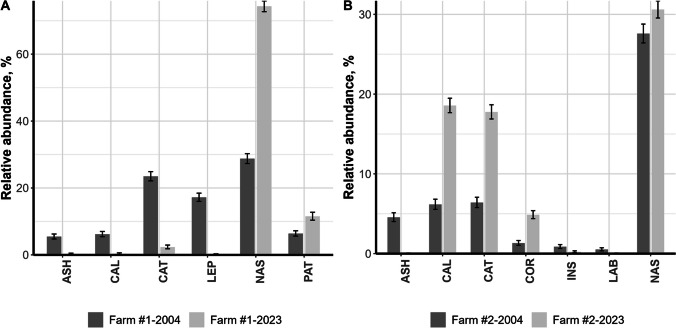
Relative abundance (%) for separate strongylid species for Farm #1 (**A**) and Farm #2 (**B**). Only species that substantially changed their relative abundance between 2004 and 2023 and were present in both years are included. The confidence interval limits for each species are shown with error bars. Abbreviations: ASH, *Cylicocyclus ashworthi*; NAS, *C. nassatus*; LEP, *C. leptostomum*; CAL, *Cylicostephanus calicatus*; CAT, *Cyathostomum catinatum*; PAT, *C. pateratum*; COR, *Coronocyclus corontus*; LAB, *C. labiatus*

The relative abundance (RA) of individual species in the community also changed significantly for some species over two decades of regular anthelmintic treatments (Fig. 1A, B), while for the other species, RA was pretty stable. Moreover, changes in the RA did not completely coincide for certain cyathostomin species on Farms #1 and Farm #2 (Table 1, Fig. 1A, B).

### Changes in the strongylid communities on two farms

**Fig. 2:**
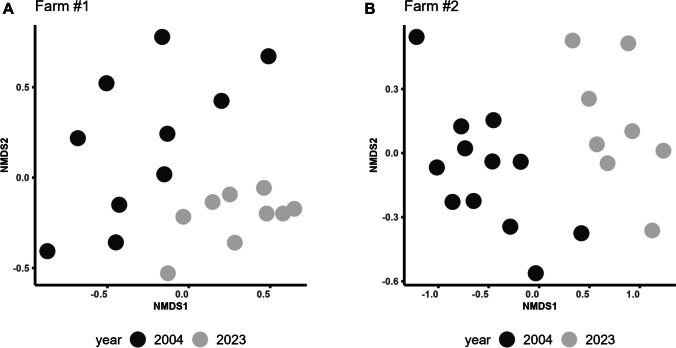
nMDS ordinations of Bray–Curtis dissimilarities of strongylid abundance matrices from Farm #1 (**A**) and Farm #2 (**B**). Data were first square-root-transformed and then Wisconsin-transformed. Separate ordinations were performed for each of the two farms. Each dot represents an individual horse (an infracommunity of strongylids), with the distance between dots indicating the degree of dissimilarity between infracommunities

Changes in the strongylid infracommunities over two decades were observed on both farms. Visualization of the similarity between strongylid infracommunities using the nMDS demonstrated two distinct groups of 2004 and 2023 on both farms (Fig. 2).

Based on the SIMPER analysis, six cyathostomin species were found to contribute most (cumulative contribution 75.87%) into the dissimilarity of strongylid communities on Farm #1: *C. nassatus* (29.90%), *C. catinatum* (13.22%), *C. pateratum* (9.57%), *C. leptostomum* (9.48%), *C. calicatus* (7.33%) and *C. ashworthi* (6.37%). On Farm #2 seven species: *C. catinatum* (12.66%), *C. calicatus* (12.24%), *C. nassatus* (11.08%), *C. leptostomum* (10.93%), *C. longibursatus* (10.20%), *C. minutus* (7.26%), and *C. coronatus* (6.55%) contributed most (cumulative contribution 70.91%) into the dissimilarity of strongylid communities between 2004 and 2023.

### Changes in the strongylid community diversity and structure

Cyathostomins predominated in the strongylid communities on both farms in 2004 and 2023. According to the data collected in 2023, on Farm #1, four cyathostomin species (*C. catinatum*, *C. nassatus*, *C. longibursatus*, and *C. goldi*) with *P* > 80% dominated in the community; the total proportion of four dominant species in the strongylid community was 86.0%. On Farm #2, five species (*C. catinatum*, *C. nassatus*, *C. calicatus*, *C. minutus*, and *C. goldi*) were dominant (*P* > 80%); the total proportion of dominant species was 77.6%.

In 2004, the number of dominant (with *P* > 80%) species was higher on both farms; on Farm #1, six cyathostomin species (*C. catinatum*, *C. pateratum*, *C. nassatus*, *C. ashworthi*, *C. longibursatus*, and *C. goldi*) dominated the community (*P* > 80%); their total proportion in the community was 71.3%. On Farm #2, ten cyathostomin species (C*. catinatum*, *C. nassatus*, *C. ashworthi*, *C. coronatus*, *C. leptostomus*, *C. calicatus*, *C. labratus*, *C. minutus*, *C. longibursatus*, and *C. goldi*) were dominant; their total proportion in the community was 94.8%.

Margalef’s species richness index decreased significantly on both farms (Table 2) which was associated with a decrease in the strongylid species number on the farm as well as the number of species parasitized per horse over two decades. Pielou’s evenness index on Farm #1 decreased significantly indicating the uneven distribution of species. On the contrary, on Farm #2, the Pielou’s index slightly increased; thus, fewer species composed of the strongylid community in 2023 were distributed more evenly. One cyathostomin species, *C. nassatus*, dominated the strongylid communities on both farms over two decades. On Farm #1, the Berger-Parker dominance index for *C. nassatus* dramatically increased from 28.8 in 2004 to 74.4 in 2023; during the same period, on Farm #2, the Berger-Parker index slightly increased from 28.6 in 2004 to 30.6 in 2023. Shannon’s and Simpson’s diversity indexes decreased on both farms; on Farm #1, the diversity decreased more than on Farm #2 (Table 2).

**Table 2:** Characterization of strongylid communities on Farm #1 and Farm #2 in 2004 and 2023

	Farm #1		Farm #2	
	2004	2023	2004	2023
Number of species	19	12	21	9
Number of specimens	3,612	2,806	5,508	7,075
Margalef species richness index	2.441	1.385	2.322	1.015
Pielou evenness index	0.646	0.391	0.716	0.748
Berger-Parker dominance index/dominant species	28.8 */ C. nassatus*	74.4 / *C. nassatus*	27.6 */ C. nassatus*	30.6 */ C. nassatus*
Shannon diversity index	1.965	0.972	2.179	1.722
Simpson diversity index	0.817	0.429	0.844	0.799

**Fig. 3:**
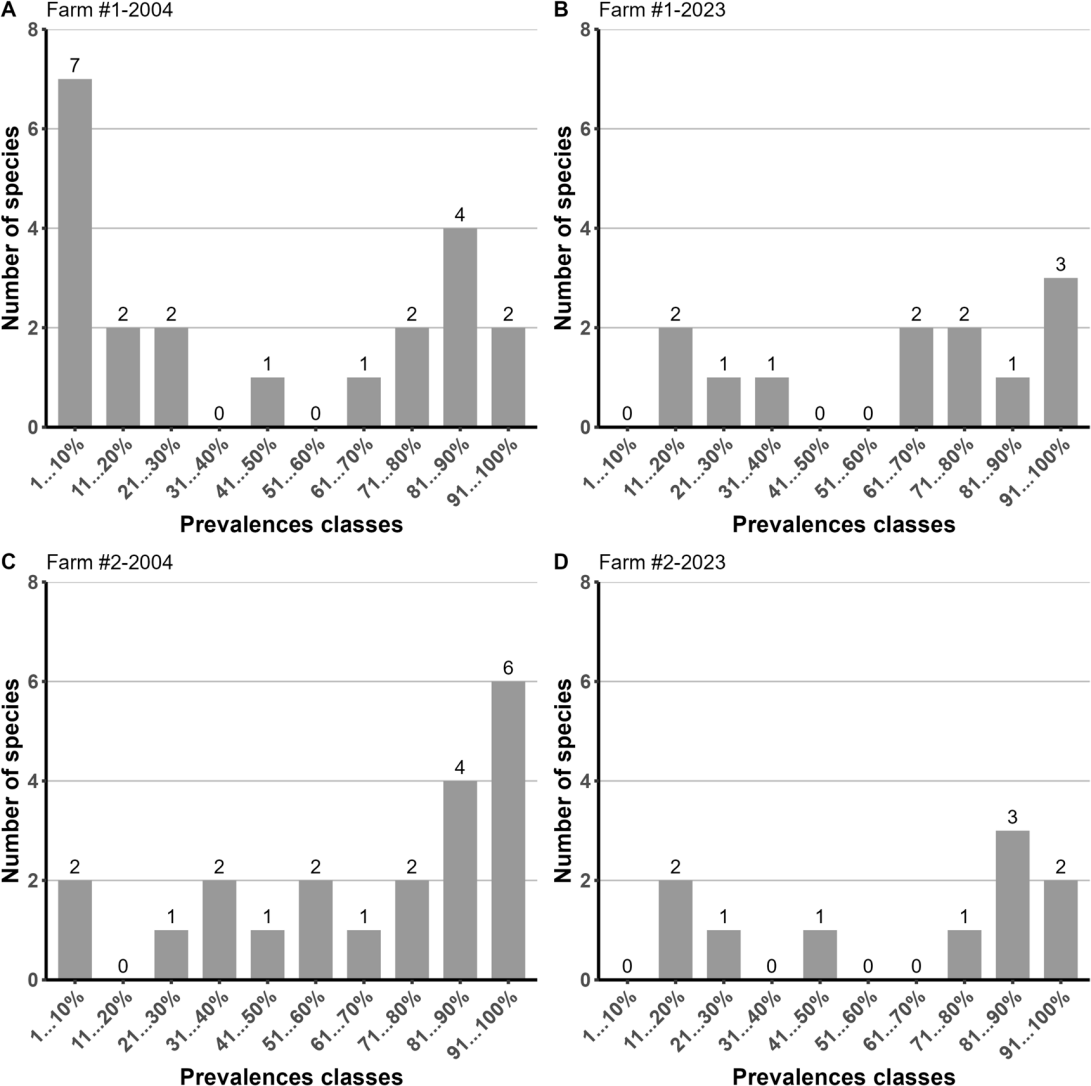
Distribution of strongylids species found on Farm #1 in 2004 (**A**) and 2023 (**B**) and on Farm #2 in 2004 (**C**) and 2023 (**D**) in ten prevalence classes

The distribution of strongylid species found in ten prevalence classes did not show substantial changes in the general structure of the strongylid communities on both farms after two decades of anthelmintic treatments (Fig. 3). Multimodal structures of the strongylid community with dominant (*P* > 80%), subdominant (*P* > 50–80%), background (*P* > 20–50%), and rare (*P* ≤ 20%) species were observed in all samples. However, in 2023, the strongylid communities on both farms (Fig. 3 B and D) showed a trend of gradually transforming from multimodal to bimodal (core–satellite mode) — the “middle sector” of species with *P* = 30–70% gradually disappeared from the communities.

## Discussion

This paper presents the original results of a study of changes in the strongylid community in domestic horses following two decades of exposure to regular anthelmintic treatments. Frequent anthelmintic treatments are known to influence the intestinal parasite community in horses; the prevalence of highly pathogenic large strongylids (*Strongylus* spp., *Triodontophorus* spp., etc.) has significantly decreased over the past decades due to the use of highly effective anthelmintics, resulting in cyathostomins becoming the most prevalent horse parasites worldwide (Herd 1990; Osterman Lind et al. 1999; Hinney et al. 2011; Studzińska et al. 2012; Pilo et al. 2012; Kuzmina et al. 2016; Lyons et al. 2018; Sallé et al. 2018; Bellaw and Nielsen 2020; Jürgenschellert et al. 2022; Abbas et al. 2024a). At the same time, any experimental studies to assess the effect of BZ and ML anthelmintics on the species composition of the strongylid community have not been carried out due to the complexity of planning such long-term studies. DNA metabarcoding technique has been developed to identify the presence of separate strongylid species in horse fecal samples (Poissant et al. 2021; Sargison et al. 2022; Courtot et al. 2023; Halvarsson et al. 2024; Abbas et al. 2024b); however, this method does not allow the accurate estimation of the intensity or relative abundance of the individual species within the community. Moreover, GenBank currently lacks representative sequences for each known species of equine strongylids. Therefore, using classical parasitological methods of collecting and morphological identification of strongylids remains necessary to study their community.

This study has limitations due to the small number of farms included in the research and the small number of horses surveyed on each farm, which were smaller compared to previous studies of strongylid communities (Nielsen et al. 2010; Kuzmina et al. 2016; Sallé et al. 2018, Boisseau et al. 2023). Of the 11 farms surveyed in 2004–2006 (Kuzmina and Kharchenko 2008), we could repeat the strongylid community study only on two farms due to various circumstances beyond our control. Therefore, the data collected and analyzed in this study are unique. Although despite the relatively large number of horses kept on these two farms in 2023 (40 and 18 horses), only a small number of animals (with EPG > 200) could be selected for the study because of the low level of strongylid infection (EPG < 200) for most horses at both farms.

This reduction in strongylid infection levels was consistent with the results of monitoring studies performed in recent years, which have shown that the level of strongylids infection in domestic horses decreased due to the frequent use of anthelmintics (Nielsen et al. 2021, 2024). The number of animals with no strongylid egg in fecal samples (EPG = 0) accounted for up to 50–60% of horses in a studied population (Nielsen et al. 2024); at the same time, the “80/20 rule,” while 20% of mature horses shed 80% of the total number of eggs, remained stable (Relf et al. 2013; Wood et al. 2013; Nielsen et al. 2018, 2024). Nevertheless, this low proportion of infected horses allows strongylids to maintain their transmission, contaminate the environment (pastures and paddocks), and even develop resistance to anthelmintic drugs. Thus, there is a need to thoroughly study strongylid species that have effective mechanisms to survive regular treatments with highly effective anthelmintics. Furthermore, the trend will probably also be a limitation for future research on strongylid communities in horses.

Overall, 21 strongylid species, including six species of large strongylids and 15 species of cyathostomins, were found in samples in 2004, which served as reference points in this research. Similar results were obtained in surveys of helminth communities of domestic horses in other countries at that time (Cirak et al. 1996; Silva et al. 1999; Lyons et al. 2001; Chapman et al. 2002; Anjos and Rodrigues 2003; Osterman Lind et al. 2003, 2007; Kuzmina et al. 2005; Kornaś et al. 2009). Based on data collected from the same farms two decades later, we revealed a substantial decline in the richness of strongylid communities, primarily due to the disappearance of large strongylids and rare species of cyathostomins. This finding was consistent with the general trend observed worldwide in recent decades (Sallé et al. 2020; Courtot et al. 2023; Mattews et al. 2023).

A comparison of strongylid infracommunities on both farms visualized by the nMDS demonstrated obvious changes over two decades. According to information received from horse owners, horse management practices (horse-keeping conditions, stocking density, stable cleaning, access to pasture, etc.), have not changed over the last two decades, except for the deworming frequency. Thus, all changes in the strongylid community structure observed in this study are associated exclusively with anthelmintic treatments. Besides the disappearance of large strongylids, we revealed an interesting trend in the disappearance of rare (*P* < 20%) cyathostomin species such as *C. bidentatus*, *C. ultrajectinus*, *P. mettami*, *P. poculatum*, *C. elongatus*. The prevalence of background species (P > 20–50%) such as *C. labiatus*, *C. labratus*, and *C. insigne* decreased dramatically, and these species became rare. At the same time, dominant (*P* > 80%) and subdominant (*P* > 50–80%) species virtually did not change their prevalence; moreover, their proportion in the strongylid community increased. We observed this trend earlier in the strongylid communities in domestic horses from farms with different anthelmintic treatment regimens (Kuzmina et al. 2011, 2016) and in other domestic and wild equids (Kuzmina et al. 2007, 2013, 2020; Slivinska et al. 2016). We believe that the changes are associated with the specific response of individual cyathostomin species to frequent deworming. Apparently, the species that successfully survived two decades of regular deworming with highly effective ML anthelmintics do have unique physiological and genetic mechanisms. This assumption prompted us to elucidate the contribution of separate species in these strongylid community alterations.

SIMPER analysis showed that 6–7 cyathostomin species made the greatest contribution to the dissimilarity of strongylid communities on the farms examined. *Cylicocyclus nassatus* contributed the most to the dissimilarity of strongylid communities on Farm #1 and was one of the three most important species determining dissimilarity on Farm #2. Also, in this study, we recorded a significant increase in the dominance of *C. nassatus* in the strongylid communities. This phenomenon was most clearly demonstrated on Farm #1, where the Berger-Parker dominance index for *C. nassatus* dramatically increased from 28.8 in 2004 to 74.4 in 2023. Thus, after two decades of frequent use of ML drugs, one “super-dominant” species accounted for almost three-quarters of nematodes parasitizing the horses. In our previous studies, we observed an increase in the dominance of this species in the strongylid communities of domestic and wild equids (Kuzmina et al. 2005, 2013, 2016); however, the proportion of this species in the community did not exceed 44–45%. We believe that *C. nassatus*, which has high haplotype variability (Traversa et al. 2008), possesses sufficiently high genetic plasticity that allows this species to adapt to frequent use of anthelmintics and, probably, develop anthelmintic resistance faster than other cyathostomins. Therefore, a detailed study of this particular species will probably provide new information about the mechanisms of the development of anthelmintic resistance in strongylids.

The results of this study did not allow us to discuss the development of anthelmintic resistance in cyathostomin species found, although resistance to BZ and even to ML was previously observed in all these species (Tolliver et al. 1993; Lyons et al. 1996; Kuzmina and Kharchenko 2008; van Doorn et al. 2014; Kuzmina et al. 2020; Abbas et al. 2024a), since the absence of strongylid eggs in the fecal samples on the 14th day after deworming indicated the 100% efficacy of ML drug used. Therefore, we hypothesize that the physiological peculiarities of cyathostomins that ensure their survival of regular/frequent anthelmintic treatments may be associated with successful refugia and survival of these species on pasture (Hodgkinson et al. 2019) or with a shortened egg-reappearance period (Nielsen et al. 2022) and not with high egg productivity of these species, since the egg production of most species found in our studies was quite low (Kuzmina et al. 2012). Accordingly, the mechanisms of survival of certain cyathostomin species during long-term exposure to anthelmintics require further study.

In the present study, the distribution of strongylid species found in two farms into ten prevalence classes revealed a trend towards a gradual transformation of the strongylid community structure from multimodal to bimodal (core–satellite mode), with background species with a prevalence of 20–50% gradually disappearing from the community. A similar trend in the strongylid community structure was previously observed in equids subjected to regular or frequent deworming (Bucknell et al. 1996; Kuzmina and Kharchenko 2008; Kuzmina et al. 2012, 2011, 2020; Slivinska et al. 2016). We predict that frequent deworming of horses using ML anthelmintics on these particular farms in the future will lead to the complete disappearance of the background category of species, which will either become rare or completely disappear from the community. Thus, the results of the present study not only indicated the presence of certain cyathostomin species with potential mechanisms for the development of anthelmintic resistance but also outlined trends in the change in the strongylid community structure in horses caused by frequent deworming.

## Supplementary Information

Below is the link to the electronic supplementary material.

Supplementary file1 (PDF 183 KB)

## Data Availability

No datasets were generated or analyzed during the current study.
