# Supplementary material for: Changes in equine strongylid communities after two decades of annual anthelmintic treatments at the farm level
Source: Parasitol Res. 2024 Nov 25;123(11):394. doi: 10.1007/s00436-024-08417-5 (PMC11588933; doi:10.1007/s00436-024-08417-5)
Supplement: Supplementary file 1 — Supplementary file1 (PDF 183 KB) [file 436_2024_8417_MOESM1_ESM.pdf]

Supplement 1.

Table. Nematode count and number of strongylid species (species richness) in horses from Farms #1 and #2 examined in 2004 and 2023.

Farm/Horse	year	Nematode count	Strongylid species richness
Farm #1/H-1-04	2004	391	15
Farm #1/H-2-04	2004	273	12
Farm #1/H-3-04	2004	1,084	19
Farm #1/H-4-04	2004	58	13
Farm #1/H-5-04	2004	876	18
Farm #1/H-6-04	2004	794	15
Farm #1/H-7-04	2004	304	15
Farm #1/H-8-04	2004	450	15
Farm #1/H-9-04	2004	555	16
Farm #1/H-10-04	2004	271	9
Farm #1/H-11-04	2004	203	12
Farm #1/H-12-04	2004	249	7
Farm #1/H-1’-23	2023	46	4
Farm #1/H-2’-23	2023	203	6
Farm #1/H-3’-23	2023	379	4
Farm #1/H-4’-23	2023	945	8
Farm #1/H-5’-23	2023	647	8
Farm #1/H-6’-23	2023	1,884	7
Farm #1/H-7’-23	2023	1,424	8
Farm #1/H-8’-23	2023	1,547	6
Farm #2/H-1-04	2004	131	11
Farm #2/H-2-04	2004	61	10
Farm #2/H-3-04	2004	113	6
Farm #2/H-4-04	2004	66	11

Farm/Horse	year	Nematode count	Strongylid species richness
Farm #2/H-5-04	2004	592	10
Farm #2/H-6-04	2004	219	8
Farm #2/H-7-04	2004	634	12
Farm #2/H-8-04	2004	694	15
Farm #2/H-9-04	2004	1,040	10
Farm #2/H-10-04	2004	62	8
Farm #2/H-1’-23	2023	345	9
Farm #2/H-2’-23	2023	193	10
Farm #2/H-3’-23	2023	341	7
Farm #2/H-4’-23	2023	366	7
Farm #2/H-5’-23	2023	192	6
Farm #2/H-6’-23	2023	482	6
Farm #2/H-7’-23	2023	222	7
Farm #2/H-8’-23	2023	264	7
Farm #2/H-9’-23	2023	401	9

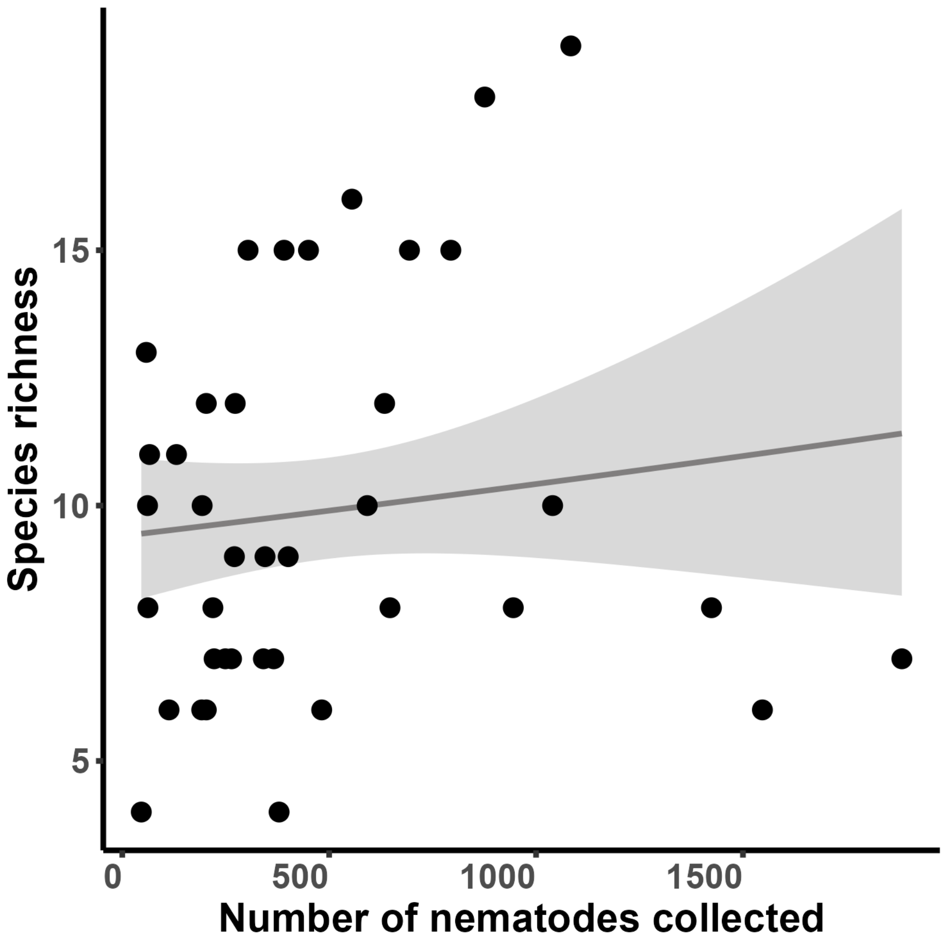

Figure. Impact of sampling effort on observed species richness in nematode infracommunities.
